# Supplementary figures and images for: Transcriptome and structure analysis in root of Casuarina equisetifolia under NaCl treatment
Source: PeerJ. 2021 Sep 22;9:e12133. doi: 10.7717/peerj.12133 (PMC8464194; doi:10.7717/peerj.12133)

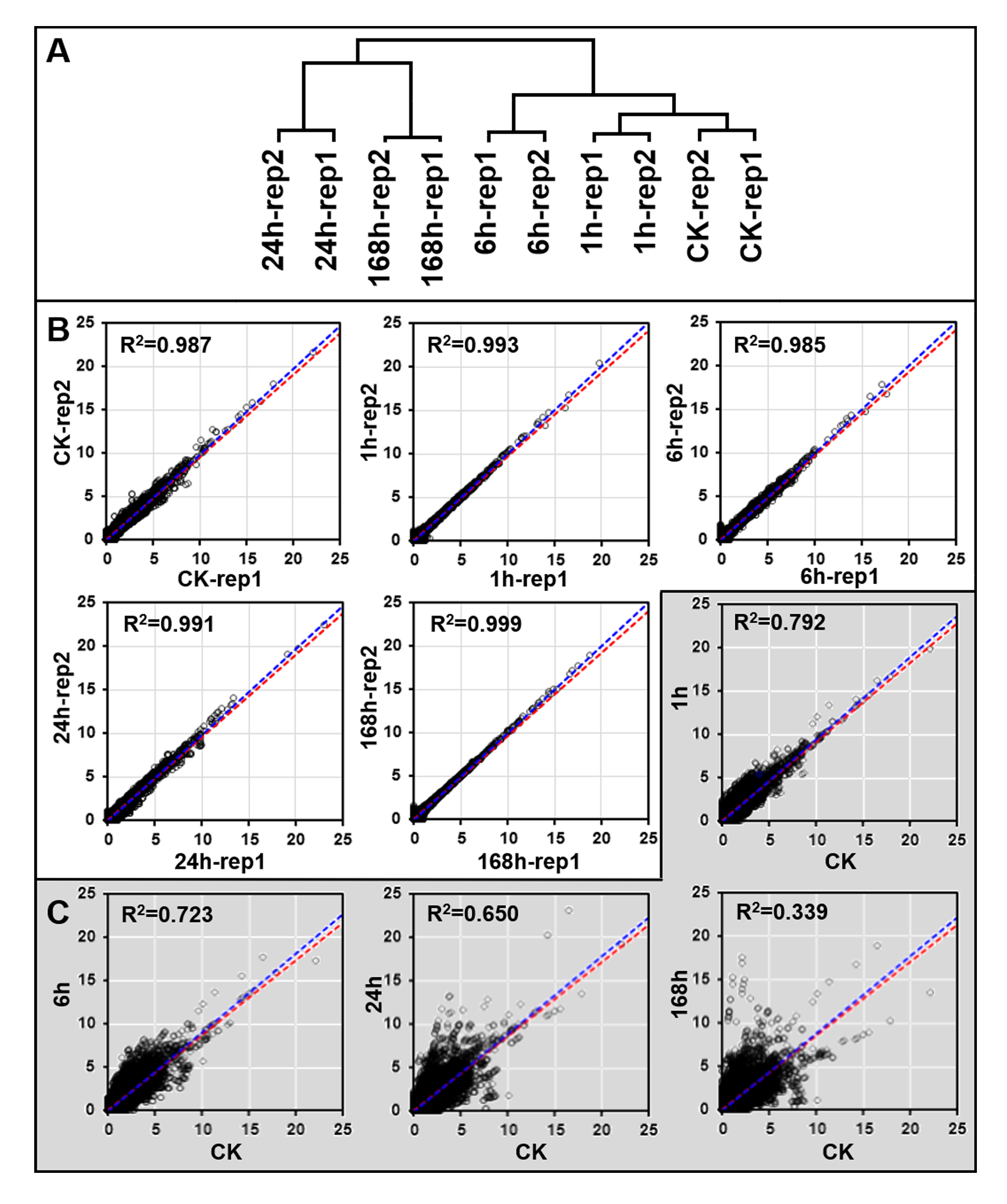

Supplement: Supplemental Information 1 — (A) All samples were cluster. (B) Pair-wise Pearson’s correlations of expression values between biological replicates. (C) The correlation of expression values between the treatment and control samples. [file peerj-09-12133-s001.jpg]

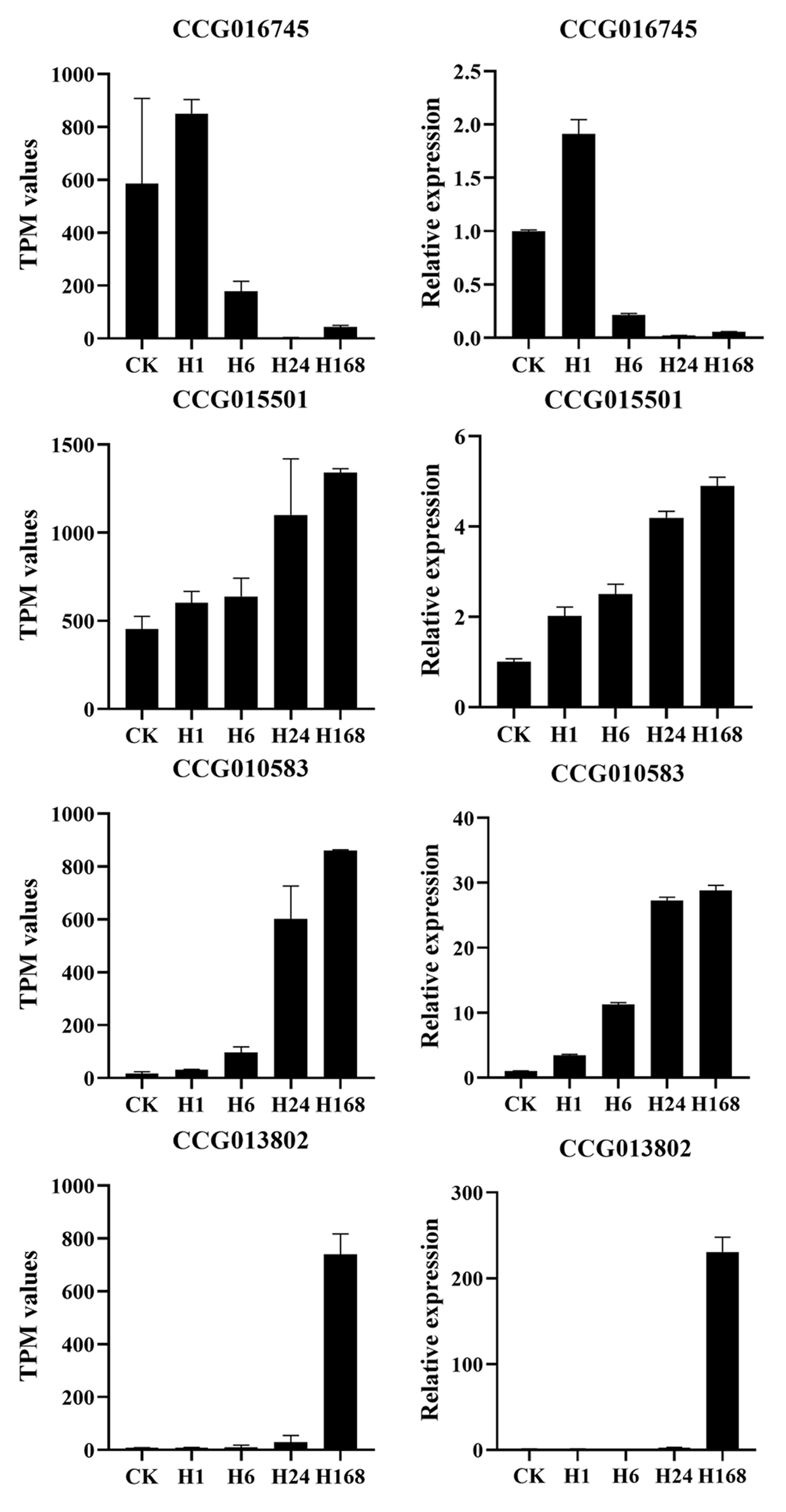

Supplement: Supplemental Information 2 — Left panel: The relative expression levels of target genes from qRT-PCR results, and TPM values were acquired by RNA-seq. Right panel: Expression profiles of some DEGs, and the heatmap was constructed by R package (Pheatmap). [file peerj-09-12133-s002.png]
